# Impact of Periampullary Diverticula on Bile Duct Stones and Ampullary Carcinoma

**DOI:** 10.5005/jp-journals-10018-1162

**Published:** 2016-07-09

**Authors:** Necati Örmeci, Xheni Deda, Çağdaş Kalkan, Ali Emrehan Tüzün, Fatih Karakaya, Abdulkadir Dökmeci, D Kadir Bahar, Hasan Özkan, Ramazan İdilman, Kubilay Çınar

**Affiliations:** 1 Department of Gastroenterology, Ankara University School of Medicine, Ankara, Turkey

**Keywords:** Ampullary carcinoma, Bile duct stones, ERCP, Periampullary diverticula.

## Abstract

**Introduction:**

Periampullary diverticula (PD) is caused by extraluminal pouching of duodenal mucosa. Using a very common endoscopic procedure to diagnose or treat gastrointestinal disorders, we encountered duodenal diverticulum.

**Materials and methods:**

This is a retrospective, single-center study. Three thousand and sixteen patients on whom endoscopic retrograde cholangiopancreatography (ERCP) was performed at Ankara University Medical School, Department of Gastroenterology, from June 2009 to June 2014 were included to the study.

**Results:**

Hundred and thirty patients (males 65, females 65) among the 3,016 had PD. Two hundred and sixty patients without diverticulum were randomly chosen from the 3,016 patients, as a control group [121 (47%) females, 139 (53%) males]. There was no statistical difference between the two groups. The mean age of the patients with PD was 69.9 years, while the mean age was 62.3 years for patients without PD (p < 0.001). Incidence for PD was 4.6%. The papilla of Vater was located in the inter-diverticular area (Type 1) in 9 patients (8.3%), at the edge of the diverticulum (Type 2) in 31 patients (28.4%), and at a distance of 2 to 3 cm from the papilla (Type 3) in 69 patients (63.3%).

**Discussion:**

Although numerically more common bile duct stones occurred in patients with PD compared to those without PD, there was no statistical difference between the two groups. The rate of pancreato-biliary carcinomas was higher in patients without diverticulum. Cannulation was successful in both groups at the rate of 97.6 and 92% respectively, but cannulation failed more often in patients without PD. Duodenal perforation occurred in one patient with PD. Bleeding after sphincterotomy occurred in two patients without PD.

**How to cite this article:**

Örmeci N, Deda X, Kalkan Ç, Tüzün AE, Karakaya F, Dökmeci A, Bahar DK, Özkan H, İdilman R, Çınar K. Impact of Periampullary Diverticula on Bile Duct Stones and Ampullary Carcinoma. Euroasian J Hepato-Gastroenterol 2016;6(1):31-34.

## INTRODUCTION

Periampullary diverticula (PD) is caused due to extralu-minal pouching of duodenal mucosa. Using a very common endoscopic procedure in order to diagnose or treat gastrointestinal disorders, we encountered several cases of duodenal diverticulum. Several duodenopancreatic diseases, such as common bile duct stones, cholangitis, and pancreatitis are seen together with duodenal diverticulum. The aim of the study is to clarify the relationship of duodenal diverticulum with related disorders, such as biliary stone diseases, duodenal diverticula and pancreaticobiliary malignancy and duodenal diverticulum. We also checked the correlation between diverticulum localizations and the encountering diseases and assessed if there were any differences in the endoscopic retrograde cholangiopancreatography (ERCP) cannulation between patients with duodenal diverticula and control cases. Finally, we evaluated whether there were any differences in the post-ERCP complications between patients with or without duodenal diverticulum.

## MATERIALS AND METHODS

This was a retrospective, single-center study. Three thousand and sixteen patients on whom ERCP was performed at the Department of Gastroenterology, Ankara University Medical School, Ankara, Turkey, from June 2009 to June 2014 were included in the study. Video-bands and reports of the 3,016 patients were analyzed in terms of definite diagnosis, the presence or absence of duodenal diverticulum, the size of the diverticulum, localizations, such as inside of diverticulum or upper or lower right or left edge of the diverticulum, distance of 2 to 3 cm from the Vater of papilla, successful cannulation rate, and post-ERCP complication rate.

Hundred and thirty patients had duodenal diverticulum. Two hundred and sixty patients were randomly chosen among the patients who did not have duodenal diverticulum as the control group.

## RESULTS

**Fig. 1:**
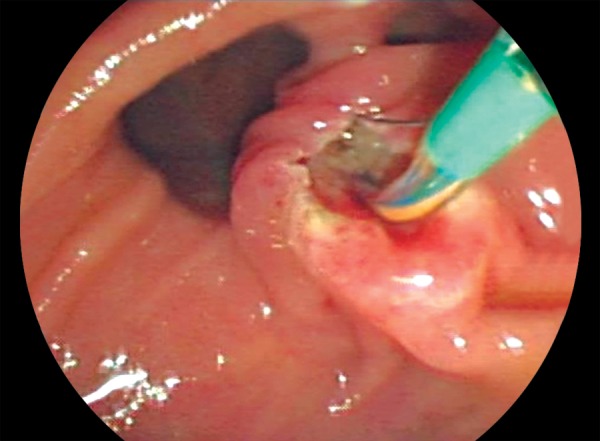
The papilla of Vater located in the interdiverticular area (Type 1) is seen during the papillotomy

Hundred and thirty patients (males 65, females 65) among 3,016 patients had PD. Two hundred and sixty patients without diverticulum were randomly chosen from those 3,016 patients as a control group [121 (47%) females, 139 (53%) males]. There was no statistical difference between the two groups. The mean age of the patients with PD was 69.9 years, while the mean age was 62.3 years for patients without PD (p < 0.001). The incidence for PD was 4.6%. The papilla of Vater was located in the inter-diverticular area (Type 1) in 9 patients (8.3%; Fig. 1), at the edge of the diverticulum (Type 2) in 31 patients (28.4%), and at a distance of 2 to 3 cm from the papilla (Type 3) in 69 patients (63.3%).

**Graph 1:**
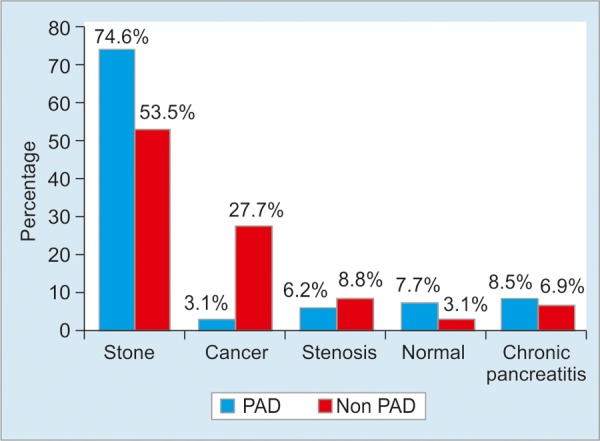
Diagnoses of patients with and without PD

Diameters of PD varied from 3 to 62 mm (mean value: 17 mm). When the patient’s age is increased, the diameters of PD also increase. Diagnoses of patients with and without PD are seen in Graph 1. Although common bile duct stones are more common in patients with PD compared to those without PD (74.6 *vs* 53.5%), there is no statistical difference between the two groups. Cancer rate for the choledocho-pancreatico-duodenal region is higher in patients without PD compared to patients with PD (27.7 *vs* 3.1% p < 0.001).

Complication rates after ERCP are seen in Table 1. There was no statistical difference in terms of acute cholangitis and acute pancreatitis between the two groups. One of the patients with PD had perforation, and two of the patients without PD had bleeding after papillotomy.

**Table 1:** **Table 1:** The complication rates after endoscopic retrograde cholangiopancreatography

		*Patients with* * periampullary* * diverticulum N (%)*		*Patients without* * periampullary* * diverticulum N (%)*	
Acute cholangitis		2 (1.5)		4 (1.5)	
Acute pancreatitis		4 (3.1)		6 (2.3)	
Perforation		1 (0.8)		–	
Bleeding		–		2 (0.8)	

The success rate for cannulation during ERCP is seen in Table 2. There was no statistical difference in terms of success rates for the first and second attempts in patients with and without PD. However, there is a statistical difference in terms of failed cannulation in one patient (0.07%) and in 19 patients (7%) (p < 0.001) with and without PD respectively.

**Table 2:** **Table 2:** Success rates for cannulation during endoscopic retrograde cholangiopancreatography

		*Patients with* * PD N (%)*		*Patients without* * PD N (%)*		*p-value*	
Cannulation in the first attempt		127 (97.6)		239 (92)			
Cannulation in the second attempt		2 (1.5)		2 (1)			
Failed cannulation		1 (0.07)		19 (7)		< 0.001	

## DISCUSSION

Periampullary diverticulum occurs as a result of defect during the fusion of foregut and midgut in embryonic life. This defect causes the decrease in the smooth muscles of duodenum.^1-3^ Acquisitioned factors, such as aging, dysfunction of Oddi sphincter, and increased intraduodenal pressure contribute to development of PD.^4^ The incidence of PD varies between 0.16 and 31.7% according to the diagnostic method.^5-9^ When the patient’s age is increased, the diameter and the occurrence of PD also increase.^10^ In our series, the incidence of PD was 4.6%, which is less than that recorded in most of the literature. The reason may be genetic factors and the low mean age in our series.

It has been shown that there are several abnormalities such as flexion, compression, and tapering of common bile duct in 31% of 107 patients with PD.^11^ Besides that, the pressure and the rate of irregular wave pattern of phasic contraction and motor abnormalities of Oddi sphincter were significantly higher in patients with PD compared to those without PD.^12,13^ Lotveit et al measured the common bile duct pressure, muscular tone, contractile activity, and total rhythmic variations of Oddi sphincter by placing the catheter into common bile duct by the way of cystic duct in eight patients with PD. The muscular tone, contractile activity, and total rhythmic variations of Oddi Sphincter during saline infusions were all significantly less in patients with PD compared to eight control cases.^14^ Skar V et al showed that there is an increase in the bacterial accommodation and in the bacterial beta-glucuronidase activity in the bile itself in patients with PD.^15,16^ Beta-glucuronidase enzyme changes the bilirubin glucuronide into free bilirubin, and by binding to calcium, the enzyme causes the common bile duct pigment stones. Another reason for the occurrence of common bile duct stones is the diverticular compression to common bile duct. All those findings may support that the reasons for the rates of common bile duct stones, acute cholangitis, and acute recurrent pancreatitis are higher in patients with PD.^17-20^ Spontaneous perforation, diverticulitis, intestinal obstruction, and increased bleeding after endoscopic sphincterotomy are reported in patients with PD compared to the control group.^7,9^

Slowness of bile in the distal common bile duct results in contacting of carcinogenic materials to the bile duct walls. This may provocate bile duct stones and/or pancreatic tumors. In our series there is a statistical difference between patients with and without PD. On the contrary, patients with PD have less carcinoma compared to control cases. This discrepancy may be because of having more indication of ERCP in patients with pancreatobiliary carcinoma.

The success rate of ERCP cannulation in patients with PD has been increasing in the experienced centers, and it varies from 85 to 98% (Divers). Katsinelos P et al reported that there is no statistical difference in terms of cannulation rate, procedural time, and procedural complication rate, but the fluoroscopy time is longer in 107 patients with PD compared to 321 control cases.^21,22^ Lobo et al mentioned that cannulation rate was 92.7% in patients without PD, but it was 62% in patients with PD (p < 0.0001).^23^

In our series, cannulation rates were 97.6 and 92% respectively in patients with and without PD. However, cannulation failed in patients without PD due to pancreatobiliary carcinoma. Complication rate were similar in patients with and without PD, and it was compatible with the literature. When the papilla of Vater is located in the diverticulum (Type I), the cannulation is more difficult and the complication rate is higher than in types 2 and 3 cases.^11,18,24,25^

## CONCLUSION

Hundred thirty patients with PD among 3,016 patients on whom ERCP was performed and 260 patients without PD who were included randomly were analyzed. The incidence of PD was 4.6%. Females and males were equal in terms of having PD. When the age was increased, the diameter of common bile duct stones and the occurrence of PD also increased. Although numerically more common bile duct stones occurred in patients with PD compared to those without PD, there were no statistical difference between the two groups. The rate of pancreatobiliary carcinomas was higher in patients without diverticulum. Cannulation was successful in both groups at the rate of 97.6 and 92% respectively, but cannulation failed more often in patients without PD. Duodenal perforation occurred in one patient with PD. Bleeding after sphincterotomy occurred in two patients without PD.
